# The Cost-of-Living Crisis and Adverse Childhood Experiences (ACEs) in Children and Adolescents

**DOI:** 10.1192/bjo.2026.11244

**Published:** 2026-06-30

**Authors:** Jasmine Fox

**Affiliations:** Mersey Care, Liverpool, United Kingdom

## Abstract

**Aims:**

Since 2022, rising living costs have intensified strain on families, leaving many families struggling to meet their basic needs. Economic instability increases the risk of adverse childhood experiences (ACEs), which are associated with long-term mental health (MH) challenges. This project aims to assess the impact of the cost-of-living crisis on children’s MH, focusing on the deprived area of Stoke-on-Trent. Following which, strategies to support children and families most affected will be explored both in terms of public health campaigns and strategies within mental health services.

**Methods:**

Initially, a literature review evaluated national trends of the impact of cost-of-living crisis on child MH. More substantial qualitative data on such themes was then developed through interviews with local general practitioners, mental health charities, schools, family hubs, and community centres to gain a better understanding of the local impact so that targeted strategies could be developed.

**Results:**

The crisis has led to children more frequently exposed to neglect and other forms of abuse, increasing ACE exposure. Local charities noted higher food bank reliance among families, and GPs reported rising malnutrition cases. Adolescents in colder homes reported worsened MH symptoms, often due to effects of overcrowding and fuel poverty, including increased witnessing of domestic violence and increased risk of chronic respiratory infections, with chronic diseases known to have long-standing mental health implications. Children from financially unstable families faced bullying due to unclean uniforms, as heating and washing were unaffordable. Limited access to transport and technology prevented families from attending MH appointments, deepening health inequalities. Activities such as sports clubs or swimming lessons were often sacrificed to protect family finances, preventing young people from attending activities known to maintain their MH, and isolating them from their peers. Local community centres found their budgets affected, with cuts to adolescent substance misuse and violence programmes, contributing to increased numbers of first-time young offenders.

**Conclusion:**

The cost-of-living crisis has amplified MH risks for many children, with more families experiencing financial insecurity and as such children experiencing ACEs. Recommendations to alleviate these effects include utilising principles of Make Every Contact Count, to identify and support struggling families as early as possible. Further considered are local programmes to expand school and community wellbeing services based on the ‘5 ways to wellbeing’ framework such as free activity programmes and social groups at family hubs. Furthermore, improving access to virtual MH appointments by supporting digital access through initiatives like ‘Tech 4 Families’.

